# Supplementary material for: Chiron3D: an interpretable deep learning framework for understanding the DNA code of chromatin looping
Source: Bioinformatics. 2026 Aug 21;42(Suppl 2):btag492. doi: 10.1093/bioinformatics/btag492 (PMC13501318; doi:10.1093/bioinformatics/btag492)
Supplement: Supplementary file 1 [file btag492_supplementary_data.pdf]

Supplementary Information

Supplementary Methods

Raw data sources We analyze CTCF HiChIP data from the Ewing sarcoma cell line A673_WT, published by Surdez et al (Surdez et al., 2021). Data were obtained from GEO (GEO accession number GSE133227). For the A673_WT cell line, there are a total of five reported replicates amounting to a total amount of read pairs of 320,219,396. For sequence coordinates, the reference genome hg19 was used. Cohesin and CTCF ChIP-seq profiles were also obtained from Surdez *et al.* (GEO: GSE133227). The A673 wildtype cohesin (SMC1A) HiChIP was obtained from Adane et al (Adane et al., 2021) (GEO: GSE165977).

Reference genome Windows of length 524,288 bp are tiled across the hg19 assembly with a stride of 50 kb. Each window later maps to a 105×105 contact matrix at 5 kb resolution. We exclude windows that cross chromosome ends or intersect with hg19 blacklist regions. This yields 59,812 candidate windows genome-wide before masking and 53,151 windows after our filtering.

Preprocessing The given CTCF HiChIP matrix was in .matrix.gz format and was converted to `.cool` using the `cooler` library in Python (Abdennur and Mirny, 2020). Here, the data for chromosome X and Y were excluded and the matrices log1p-transformed. Apart from that no additional matrix processing was applied. For the CTCF ChIP-seq data, we converted the given FASTQ files into BigWig files at a 1 base-pair resolution. The processing followed the steps outlined by Surdez et al. (Surdez et al., 2021).

Chiron3D Architecture Details We initialized the sequence backbone using a pretrained Borzoi model (Linder et al., 2025; Hingerl et al., 2025). Having this foundation model as a sequence feature extractor allowed the sequence embeddings to be rich with genomic and epigenomic features relevant to regulatory elements and long-range chromatin interactions. Given a one-hot encoded DNA-sequence as input of shape $524{,}288 \times 4$, the backbone encodes the sequence via stacked 1D convolutional blocks (batch normalization, GELU, convolution) with strided pooling, reducing the resolution to 128 bp and producing a $4{,}096 \times 1{,}536$ feature map. An eight-layer multi-head self-attention module (8 heads) is then applied. A U-Net–style decoder (Ronneberger et al., 2015) with skip connections restores the resolution to 32 bp, yielding embeddings of shape $16{,}384 \times 1{,}536$. Unlike in Borzoi, which center-crops to 196,608 bp for downstream track prediction, we retained the full 524,288 bp context and carry forward the complete $16{,}384 \times 1{,}536$ embedding to preserve long-range positional information relevant for modeling loop extrusion and boundary interactions.

We consider two adaptation regimes: (i) low-rank adaptation (LoRA) (Hu et al., 2022) applied to convolutional and linear layers as well as to the attention query and value projections (rank r=8); and (ii) a fully frozen backbone (no LoRA).

To map the 32 bp embeddings to the 105×105 contact target (525 kb at 5 kb resolution), we used three submodules. First, average pooling followed by a linear projection reduces the sequence to 105×128. Second, a transformer block stack (4 layers, 4 heads) models long-range dependencies at 5 kb resolution (Vaswani et al., 2017). Third, we formed pairwise features by concatenating position-wise embeddings for all pairs, producing a $105 \times 105 \times 256$ tensor. A decoder with five dilated residual blocks (increasing dilation) aggregates multi-scale context, and a final 1×1 convolution emits the 105×105 prediction. The pairwise feature formation and dilated-residual decoder followed the C.Origami paradigm (Tan et al., 2023), reparameterized for a 105×105 target at 5 kb resolution, with upsampling replaced by pooling. Overall, the model has 185 M frozen parameters and either 2.5 M trainable parameters (adapter head only) or 4.6 M with LoRA.

Evaluation Metrics We evaluated the predicted log-transformed contact maps using Mean Squared Error (MSE), insulation score correlations, distance-stratified correlations, and the Structural Similarity Index Measure (SSIM) (Wang et al., 2004). MSE was computed as the pixel-wise squared difference between the predicted and experimental matrices. To evaluate boundary predictions, we derived 1D insulation profiles for each map and computed their Pearson and Spearman (using scipy) correlations.

Since contact frequencies naturally decay with genomic distance, we also calculated these correlations independently for each diagonal offset. Finally, to assess 2D structural fidelity, we used SSIM (using scikit-image). By measuring local structural covariance, SSIM evaluates the sharpness of topological features, where a higher SSIM value reflects better retention of the underlying structural features in the interaction maps. The SSIM data range was dynamically set to the minimum and maximum values of each matrix.

Loop Datasets Construction For the stripe symmetry analysis, we used the data provided by Surdez *et al.* that was obtained with the Tweed Algorithm (Surdez et al., 2021). For classification, we follow the rules outlined by (Surdez et al., 2021) in their work. We calculated the ratio of the sum of the values for both stripes s_x, s_y, disregarding the 15 bins (75 kb) closest to the diagonal. Loops on the extremes of the asymmetry ratio were subsequently classified as asymmetrical. Through this, we ended up with 8,017 loop sites for CTCF HiChIP, 696 of which are asymmetrical, per our definition. Similarly, we obtained 2,251 loop sites for cohesin HiChIP, 101 of which are asymmetrical.

In order to obtain loops for the loop stability analysis, we used the FitHiChIP tool (Bhattacharyya et al., 2019), which detected 26,130 CTCF HiChIP loops. However, for most of these loops, many neighboring loops were observed. Therefore, within regions of 50,000 bp, we retained only the loop with the lowest q-value, reducing the total number of CTCF loops to 12,273. In the next step, we removed all loops that intersected with the identified loops by the Tweed Algorithm (Surdez et al., 2021), leaving us with 6,271 CTCF loops. For these loops, we checked whether at each loop boundary at least one cohesin subunit (RAD21, SMC1, STAG1, or STAG2) was present and whether the loop boundaries contained convergent CTCF motifs. This was done by using the CTCF forward and reverse PSSMs on the experimental CTCF peak regions at the loop boundaries, extracting the maximum binding affinity score. If the highest score at the left boundary corresponded to the forward CTCF motif and the highest score at the right boundary corresponded to the reverse CTCF motif, the loop was retained. The final number of CTCF loops was 1,895. Upon applying similar filtering for cohesin HiChIP loops, only 166 loops remain.

Train Split The final training/validation/test chromosome splits are: **train**: all autosomes except {5, 12, 13, 21, 2, 6, 19}, **validation**: {5, 12, 13, 21}, **test**: {2, 6, 19}.

Model Training Each data point consisted of a one-hot encoded DNA window of length $L_{\mathrm{bp}} =$ 524,288 bp from the hg19 reference genome, together with its corresponding CTCF HiChIP contact submatrix at 5 kb resolution. Windows were tiled genome-wide with a 50 kb stride, mapping to $L = 105$ bins and a target matrix $M_{raw} \in \mathbb{R}_{\geq 0}^{L \times L}$. Targets were transformed with $M = \log\big(1 + M_{\mathrm{raw}}\big)$. Training minimized the mean squared error on the log-transformed targets, $\mathcal{L}_{\mathrm{MSE}} = \frac{1}{|\mathcal{I}|} \sum_{(i,j)\in\mathcal{I}} \big(\widehat{M}_{ij} - M_{ij}\big)^2$, where $\mathcal{I}$ indexes all pairs in the $L \times L$ window, $\widehat{M}$ is the prediction, and M is the ground truth. Optimization was done using AdamW (Loshchilov and Hutter) (learning rate 5×10^{-4}, batch size 4) with mixed precision (bf16). A ReduceLROnPlateau (pyt) scheduler (factor 0.1, patience 5, minimum learning rate 1×10^{-7}) and early stopping using Pearson's correlation on the validation sets were used. Training converged in 24 hours on four RTX4090 GPUs. No test-time tuning was performed.

Adaptation of the C.Origami model We used C.Origami (Tan et al., 2023) as a strong baseline because it is state-of-the-art among contact map prediction models and easily adapts to our resolution. The original architecture predicted 10 kb Hi-C at 8,192 bp bins, upsampling a 210×210 matrix to 256×256. Our targets were 5 kb HiChIP measurements at 105×105, so we implemented the following as adaption: (i) We reduced the sequence and feature encoders by one layer. (ii) We halved the number of transformer blocks (8→4). (iii) We replaced the output upscaling with pooling to the desired 105×105 shape. We trained two C.Origami variants: DNA-only input and DNA+CTCF ChIP-seq (log-transformed) input. Because C.Origami operates on a 1 Mb field-of-view at 5 kb, its native output is 210×210. For head-to-head comparisons with Chiron3D, we center-cropped a 105×105 window and only evaluated against that.

We did not compare against the original C.Origami model, which required ATAC-seq as an additional input to avoid broader chromatin accessibility effects. Further, we also did not compare against DeepC or Orca, since these methods either require significant compute (Schwessinger et al., 2020;

Zhou, 2022) whereas Akita predicts observed-over-expected (O/E) contact maps, which are designed to emphasize structural features by regressing out the distance-decay relationship (Fudenberg et al., 2020). In contrast, Chiron3D targets CTCF HiChIP data, where the protein-mediated enrichment requires a model capable of capturing absolute interaction intensities and specific peak-to-background ratios that O/E normalization may obscure.

Attribution Scoring Nucleotide-level importance was computed with input×gradient (Shrikumar et al., 2017) in relation to the defined objectives *Asym* and *Stab*. These were then used as input for TF-MoDISco (Shrikumar et al., 2018), which identifies recurrent predictive sequence patterns directly from attribution maps rather than from sequence enrichment alone. Briefly, TF-MoDISco extracts short high-scoring subsequences ("seqlets") from regions with strong contribution, partitions them into metaclusters according to the sign of their contribution, aligns similar seqlets, and aggregates them into consolidated motif representations. Here, positive and negative metaclusters denote seqlets whose contributions increase or decrease the selected objective, respectively. For visualization, related TF-MoDISco motifs were further grouped into broader motif clusters following the grouping from (Vierstra et al., 2020), and the reported seqlet share for a given loop type and boundary denotes the fraction of seqlets in that subset assigned to the corresponding motif cluster. TF-MoDISco was applied only to boundary regions of symmetric and asymmetric loops. It was not used for stability analysis because the relevant intra-loop regions span several hundred kilobases and are therefore not well suited for localized seqlet-based motif discovery.

Edit Proposal Beyond visualization, the ratios *Asym* and *Stab* serve as scalar objectives for edit proposal: within a given loop window, we optimize candidate nucleotide edits to monotonically increase or decrease *Asym* or *Stab* as specified by the task. Concretely, to favor extrusion-like, stripe-dominated states we minimize *Stab*, whereas to promote stable loops we maximize *Stab*; stripe enhancement and reduction are achieved by maximizing and minimizing *Asym*, respectively.

Edit Design In silico edits were generated with Ledidi (Schreiber et al., 2025), , which formulates genomic edit design as an optimization problem in which the predictor is held fixed and the input sequence is modified to achieve a desired model output. Starting from the original one-hot encoded DNA sequence $X_0 \in \{0,1\}^{L_{\text{bp}} \times 4}$, Ledidi relaxes the discrete sequence to a differentiable representation $g(W) = \text{softmax}(W/\tau)$ where W is initialized from X_0 and $\tau > 0$ is the temperature. Given predictor $f(\cdot)$ and target objective value $\hat{y}$, we solve

$$\min_{W} \; \|g(W) - X_0\|_1 \; + \; \lambda \, \|f(g(W)) - \hat{y}\|_2^2,$$

where the ℓ_1 term encourages few edits and the second term drives the desired change in model output thus balancing sparsity and task specific objective. In our application, $f(\cdot)$ is the frozen Chiron3D-based downstream objective wrapper and $\hat{y}$ specifies the requested increase or decrease in *Asym* or *Stab*, depending on the task. After convergence, a greedy pruning pass removes superfluous substitutions to yield a minimal discrete edit set. Ledidi also supports input masks to disallow edits in specified regions.

Edit Labeling Edits were considered unlabeled unless they overlapped a CTCF ChIP-seq peak or exhibited a change in CTCF motif affinity. CTCF binding affinity was quantified using the PSSM derived from the JASPAR MA0139.1 CTCF frequency matrix (Rauluseviciute et al., 2024; Stormo, 2000). Let the CTCF motif length be L_C=19, and define a local sequence window of L_W=41 bp centered on each edit (20 bp upstream and 20 bp downstream). For a window sequence $x_{1:L_W}$ (uppercase $\{A, C, G, T\}$), we construct a log-odds PSSM $M \in \mathbb{R}^{4 \times L_C}$ assuming a uniform background p_b=0.25, with rows ordered as $\{A, C, G, T\}$. The log-odds score of a sequence segment s of length L_C is defined as

$$\text{score}(s; M) \; = \; \sum_{i=1}^{L_C} M_{\text{BASE2IDX}(s_i),\, i}$$

For each edit location, the maximum score is taken as the window-level CTCF affinity. The total change in PSSM is given by $\Delta\text{PSSM}_{\text{CTCF}} = \text{score}_{\text{post}} - \text{score}_{\text{pre}}$ where, $\text{score}_{\text{post}}$ is the maximum CTCF PSSM

score computed on the edited DNA sequence and $\text{score}_{\text{pre}}$ is the corresponding maximum score on the original sequence.

References

torch.optim.lr_scheduler.reducelronplateau. `https://docs.pytorch.org/docs/2.12/generated/torch.optim.lr_scheduler.ReduceLROnPlateau.html`. Accessed 17 May 2026; PyTorch v2.12.0.

N. Abdennur and L. A. Mirny. Cooler: scalable storage for hi-c data and other genomically labeled arrays. *Bioinformatics*, 36(1):311–316, 2020.

B. Adane, G. Alexe, B. K. A. Seong, D. Lu, E. E. Hwang, D. Hnisz, C. A. Lareau, L. Ross, S. Lin, F. S. D. Cruz, et al. Stag2 loss rewires oncogenic and developmental programs to promote metastasis in ewing sarcoma. *Cancer cell*, 39(6):827–844, 2021.

S. Bhattacharyya, V. Chandra, P. Vijayanand, and F. Ay. Identification of significant chromatin contacts from hichip data by fithichip. *Nature communications*, 10(1):4221, 2019.

G. Fudenberg, D. R. Kelley, and K. S. Pollard. Predicting 3d genome folding from dna sequence with akita. *Nature methods*, 17(11):1111–1117, 2020.

J. C. Hingerl, A. Karollus, and J. Gagneur. Flashzoi: an enhanced borzoi for accelerated genomic analysis. *Bioinformatics*, 41(9):btaf467, 2025.

E. J. Hu, Y. Shen, P. Wallis, Z. Allen-Zhu, Y. Li, S. Wang, L. Wang, W. Chen, et al. Lora: Low-rank adaptation of large language models. *ICLR*, 1(2):3, 2022.

J. Linder, D. Srivastava, H. Yuan, V. Agarwal, and D. R. Kelley. Predicting rna-seq coverage from dna sequence as a unifying model of gene regulation. *Nature Genetics*, 57(4):949–961, 2025.

I. Loshchilov and F. Hutter. Decoupled weight decay regularization. In *International Conference on Learning Representations*.

I. Rauluseviciute, R. Riudavets-Puig, R. Blanc-Mathieu, J. A. Castro-Mondragon, K. Ferenc, V. Kumar, R. B. Lemma, J. Lucas, J. Chèneby, D. Baranasic, et al. Jaspar 2024: 20th anniversary of the open-access database of transcription factor binding profiles. *Nucleic acids research*, 52(D1): D174–D182, 2024.

O. Ronneberger, P. Fischer, and T. Brox. U-net: Convolutional networks for biomedical image segmentation. In *International Conference on Medical image computing and computer-assisted intervention*, pages 234–241. Springer, 2015.

J. Schreiber, F. K. Lorbeer, M. Heinzl, Y. Y. Lu, A. Stark, and W. S. Noble. Programmatic design and editing of cis-regulatory elements. *bioRxiv*, pages 2025–04, 2025.

R. Schwessinger, M. Gosden, D. Downes, R. C. Brown, A. M. Oudelaar, J. Telenius, Y. W. Teh, G. Lunter, and J. R. Hughes. Deepc: predicting 3d genome folding using megabase-scale transfer learning. *Nature methods*, 17(11):1118–1124, 2020.

A. Shrikumar, P. Greenside, and A. Kundaje. Learning important features through propagating activation differences. In *International conference on machine learning*, pages 3145–3153. PMlR, 2017.

A. Shrikumar, K. Tian, Ž. Avsec, A. Shcherbina, A. Banerjee, M. Sharmin, S. Nair, and A. Kundaje. Technical note on transcription factor motif discovery from importance scores (tf-modisco) version 0.5. 6.5. *arXiv preprint arXiv:1811.00416*, 2018.

G. D. Stormo. Dna binding sites: representation and discovery. *Bioinformatics*, 16(1):16–23, 2000.

D. Surdez, S. Zaidi, S. Grossetête, K. Laud-Duval, A. S. Ferre, L. Mous, T. Vourc'h, F. Tirode, G. Pierron, V. Raynal, et al. Stag2 mutations alter ctcf-anchored loop extrusion, reduce cis-regulatory interactions and ewsr1-fli1 activity in ewing sarcoma. *Cancer cell*, 39(6):810–826, 2021.

J. Tan, N. Shenker-Tauris, J. Rodriguez-Hernaez, E. Wang, T. Sakellaropoulos, F. Boccalatte, P. Thandapani, J. Skok, I. Aifantis, D. Fenyö, et al. Cell-type-specific prediction of 3d chromatin organization enables high-throughput in silico genetic screening. *Nature biotechnology*, 41(8):1140–1150, 2023.

A. Vaswani, N. Shazeer, N. Parmar, J. Uszkoreit, L. Jones, A. N. Gomez, Ł. Kaiser, and I. Polosukhin. Attention is all you need. *Advances in neural information processing systems*, 30, 2017.

J. Vierstra, J. Lazar, R. Sandstrom, et al. Global reference mapping of human transcription factor footprints. *Nature*, 583:729–736, 2020. doi: 10.1038/s41586-020-2528-x. URL https://doi.org/10.1038/s41586-020-2528-x.

Z. Wang, A. C. Bovik, H. R. Sheikh, and E. P. Simoncelli. Image quality assessment: from error visibility to structural similarity. *IEEE transactions on image processing*, 13(4):600–612, 2004.

J. Zhou. Sequence-based modeling of three-dimensional genome architecture from kilobase to chromosome scale. *Nature genetics*, 54(5):725–734, 2022.

Supplementary Figures

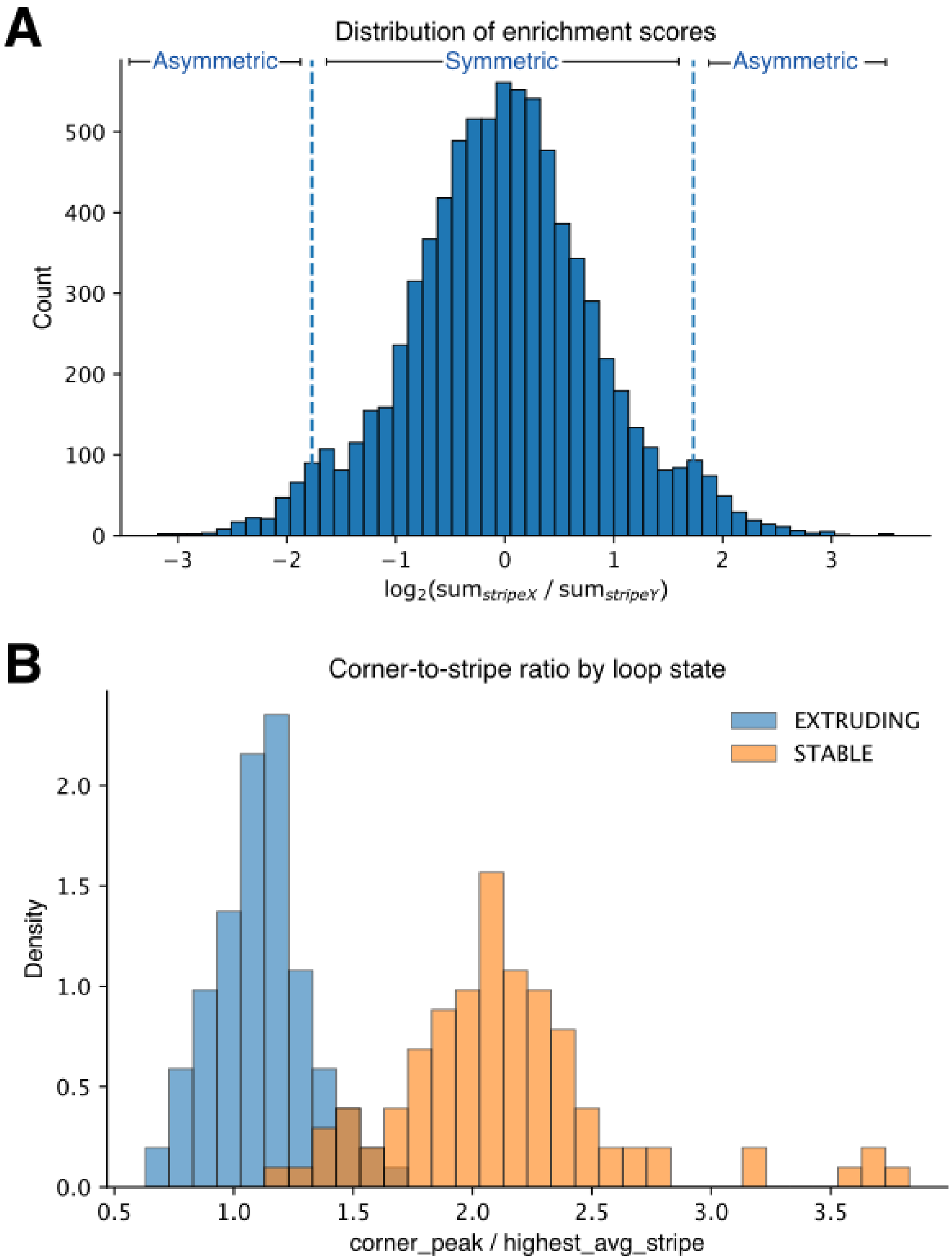

Figure S1: **Distributions of different loop types across our defined scoring metrics. A,** Distribution of Tweed-called loops across stripe ratio between X and Y stripes. The top and bottom 5% of the loops are labeled as asymmetric and otherwise symmetric. **B,** The distribution of ratio of corner interaction and highest average stripe intensity (defined by us as *Stab* score) across loops labelled as Stable and Extruding.

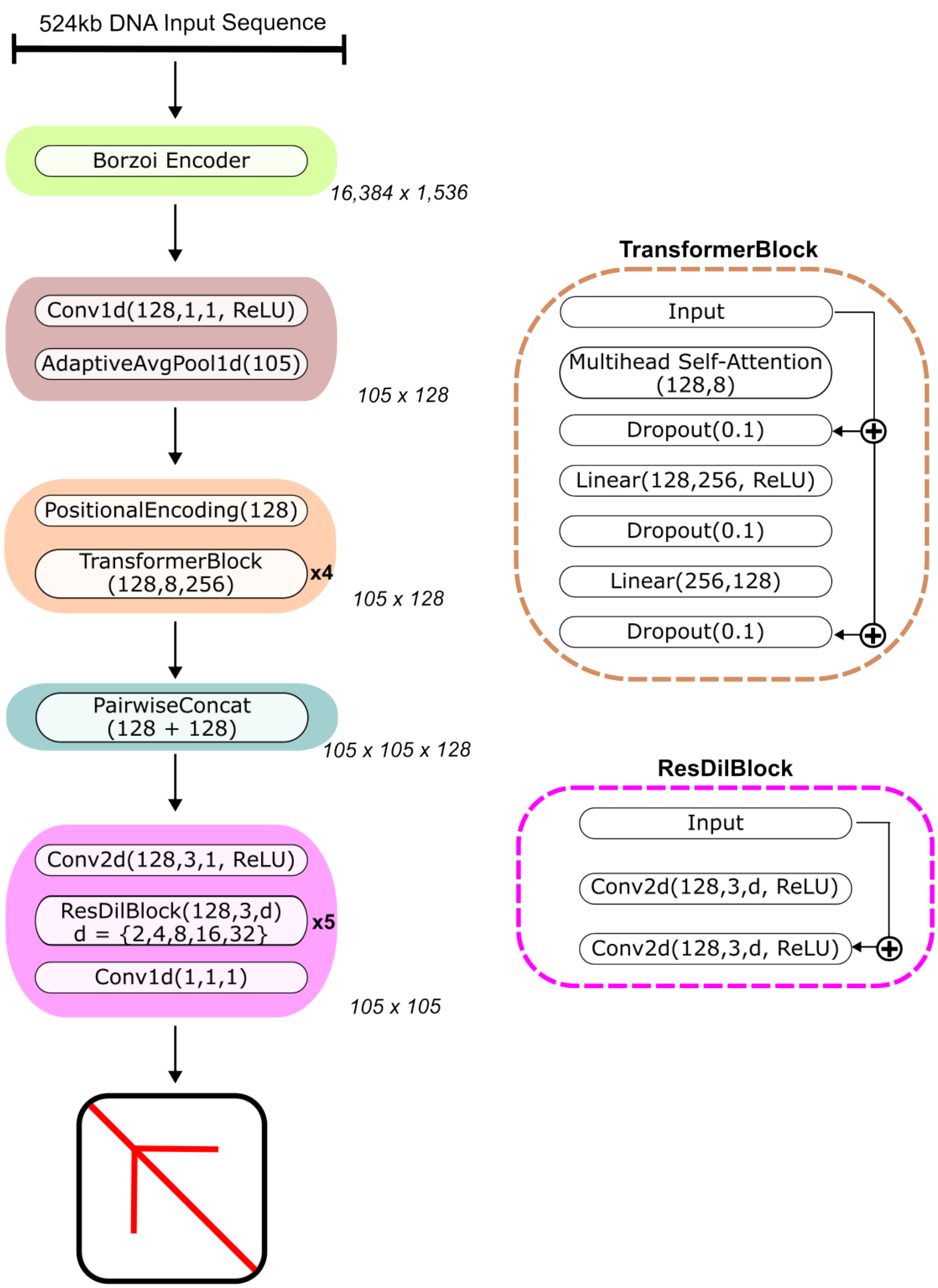

Figure S2: **Detailed architecture diagram of Chiron3D.** Chiron3D consists of a pretrained Borzoi sequence backbone that encodes the input DNA sequence into contextual embeddings capturing regulatory and long-range genomic features. These embeddings are aggregated to the target resolution and processed with transformer layers to model long-range dependencies. Pairwise combinations of positional embeddings represent potential chromatin contacts, and a dilated residual decoder predicts the final contact map. The backbone is used either frozen or adapted using low-rank adaptation (LoRA).

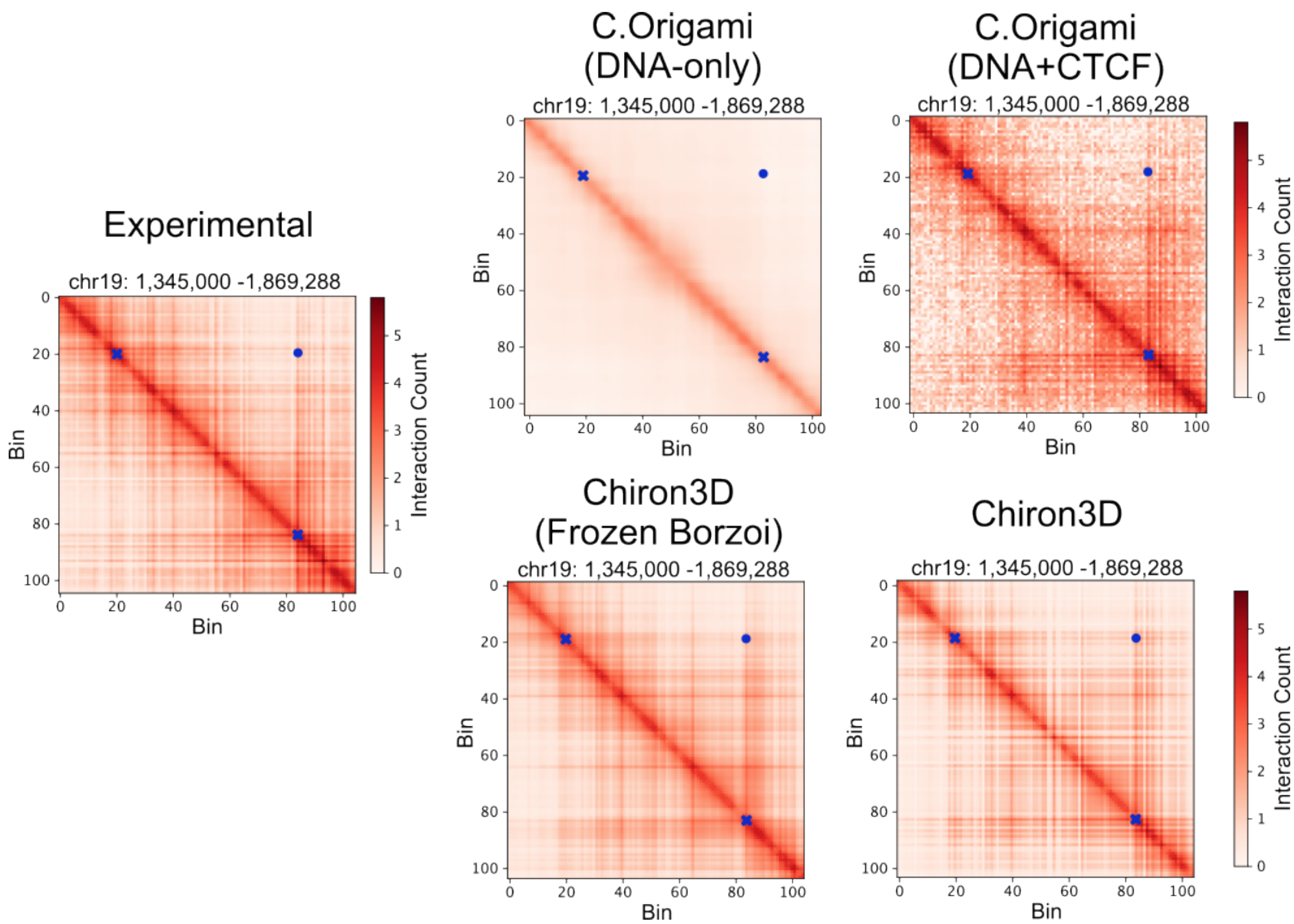

Figure S3: **Qualitative comparison between model predictions and experimental HiChIP at a representative test locus.** All models are compared on test locus chr19:1,345,000-1.869,288. The loop boundaries are marked with blue crosses whereas the corner interaction is depicted with a blue dot.

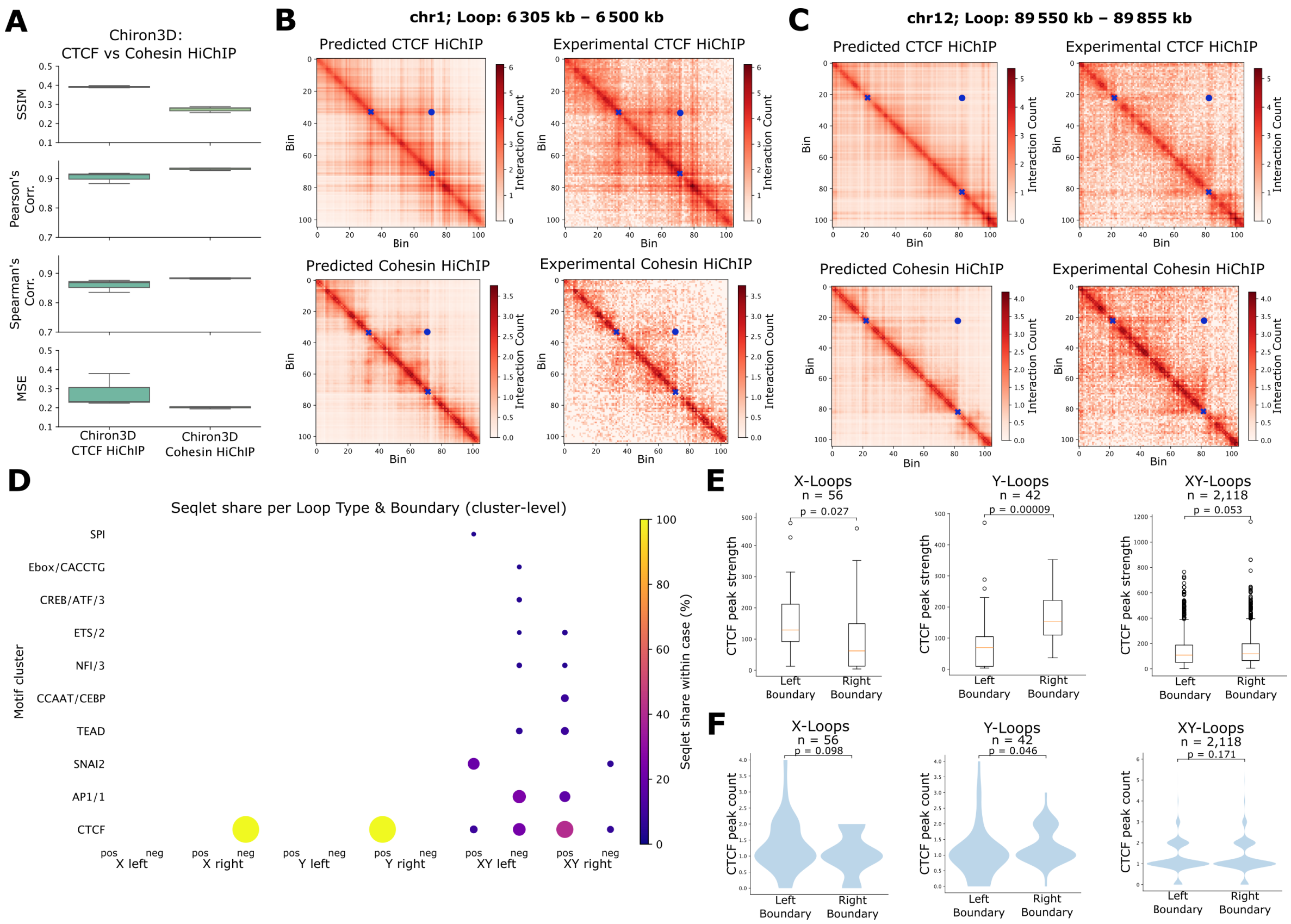

Figure S4: **CTCF is a driving factor for defining loop symmetry in cohesin HiChIP. A,** Performance of Chiron3D is compared between two training datasets: CTCF HiChIP and cohesin (SMC1A) HiChIP data. Model performance is calculated using Structural similarity index measure (SSIM), Pearson's correlation, Spearman's correlation, and mean squared error (MSE). **B-C,** Outputs of the two Chiron3D models are compared against experimental HiChIP contact maps for two representative loci. **D,** Major transcription factor motifs identified using TF-MoDISco on boundary regions of extruding cohesin loops. **E,** Measurements of CTCF ChIP-seq signal at loop boundaries of X- and Y-asymmetric cohesin loops and symmetric cohesin loops. n is the number of loops of each type. **C**, Number of CTCF ChIP-seq peaks at loop boundaries of X- and y-asymmetric cohesin loops and symmetric cohesin loops. All p-values are calculated using Wilcoxon signed-rank test.

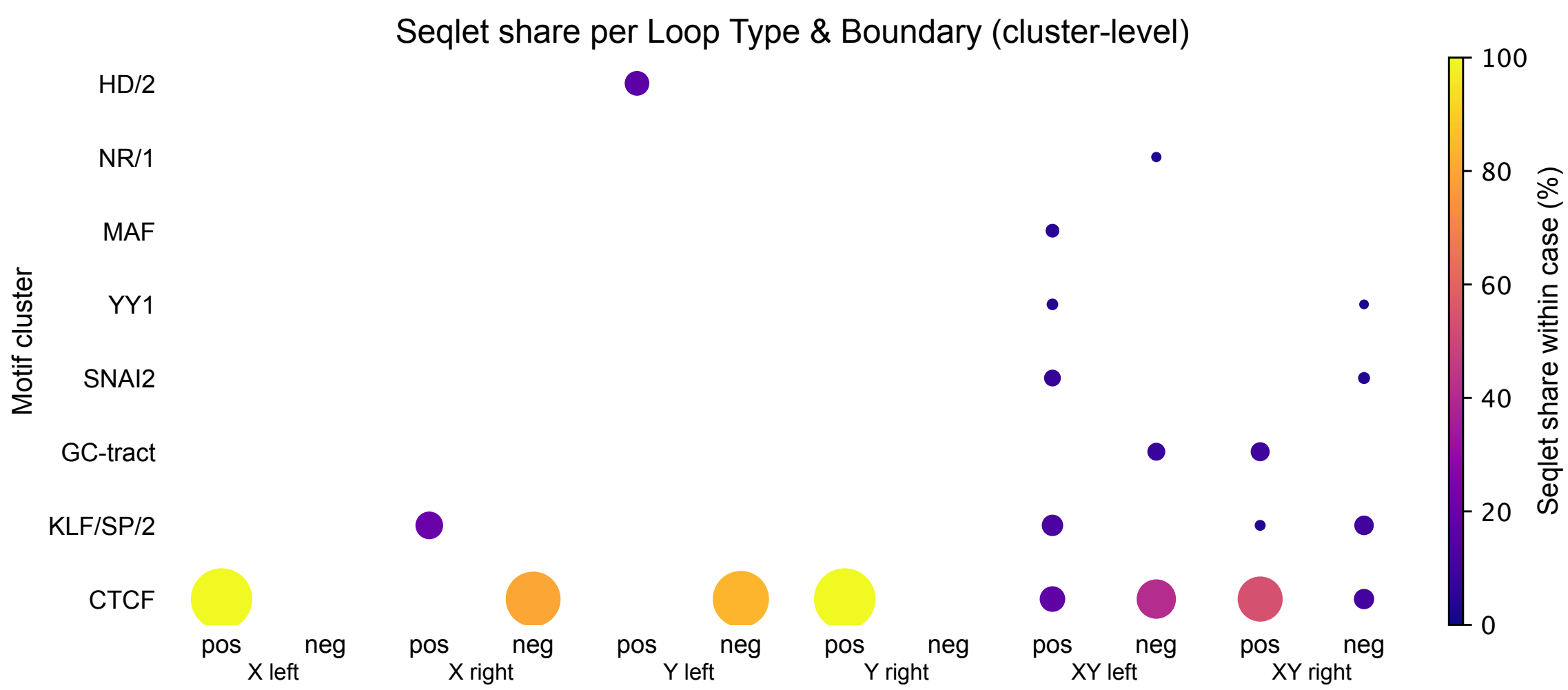

Figure S5: **CTCF as most important motif detected for loop anchorage strength.** Filtered and aggregated results from TF-MoDISco analysis on boundary regions of extruding loops.

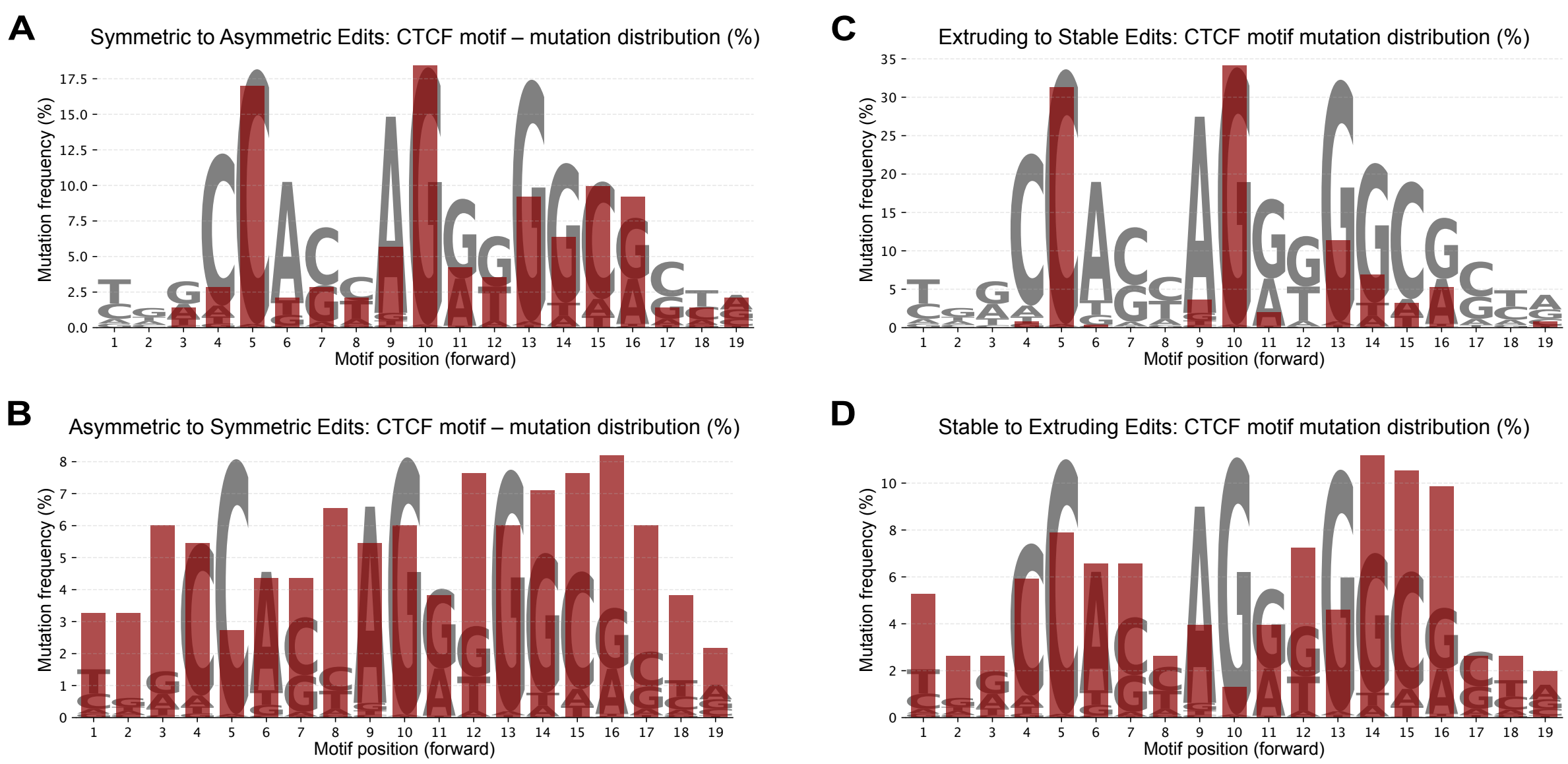

Figure S6: **Distribution of edits that fall within CTCF motif for all different objectives.** **A**, Distribution of edits within CTCF motif when proposing edits to make a loop more asymmetric. **B**, Distribution of edits within CTCF motif when proposing edits to make a loop more symmetric. **C**, Distribution of edits within CTCF motif when proposing edits to make a loop more stable. **D**, Distribution of edits within CTCF motif when proposing edits to make a loop more extruding.

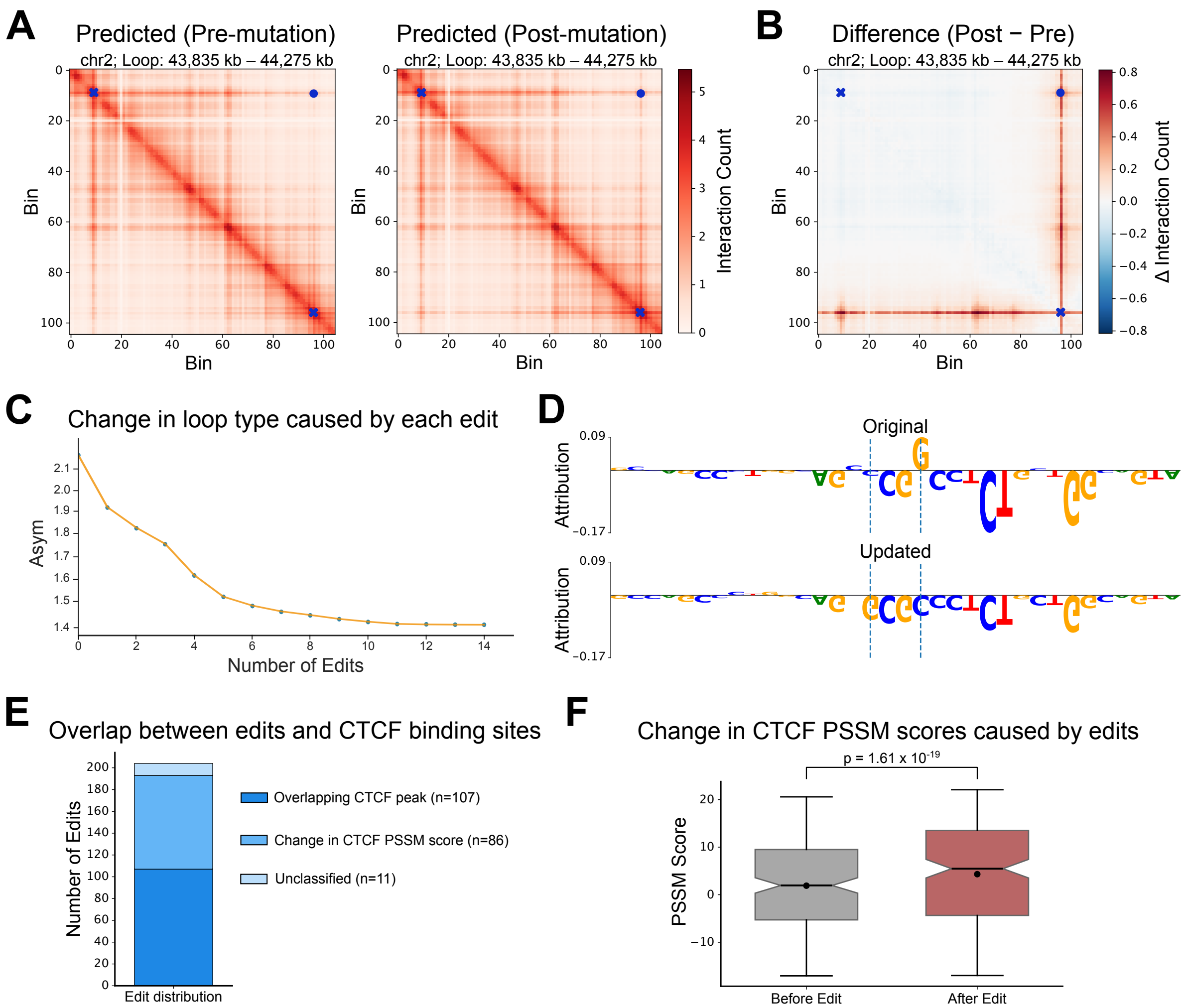

Figure S7: **Higher CTCF anchoring strength and more CTCF motifs increase anchorage probability.** **A**, Example of a region from the test set where two edits within single motif are proposed to make a asymmetric loop more symmetric. **B**, The difference in predicted contact maps caused by the proposed edit. **C**, Line plot showing change in the optimization function ($Asym$) as a function of number of proposed edits. **D**, Attribution scores before and after the edit was introduced in the sequence. **E**, Proportion of edits, proposed for symmetric loops in the test dataset, that overlap with CTCF regions. **F**, Change in CTCF PSSM scores caused by the edits across all symmetric loops in the test data. Significance is calculated using Wilcoxon signed-rank test.

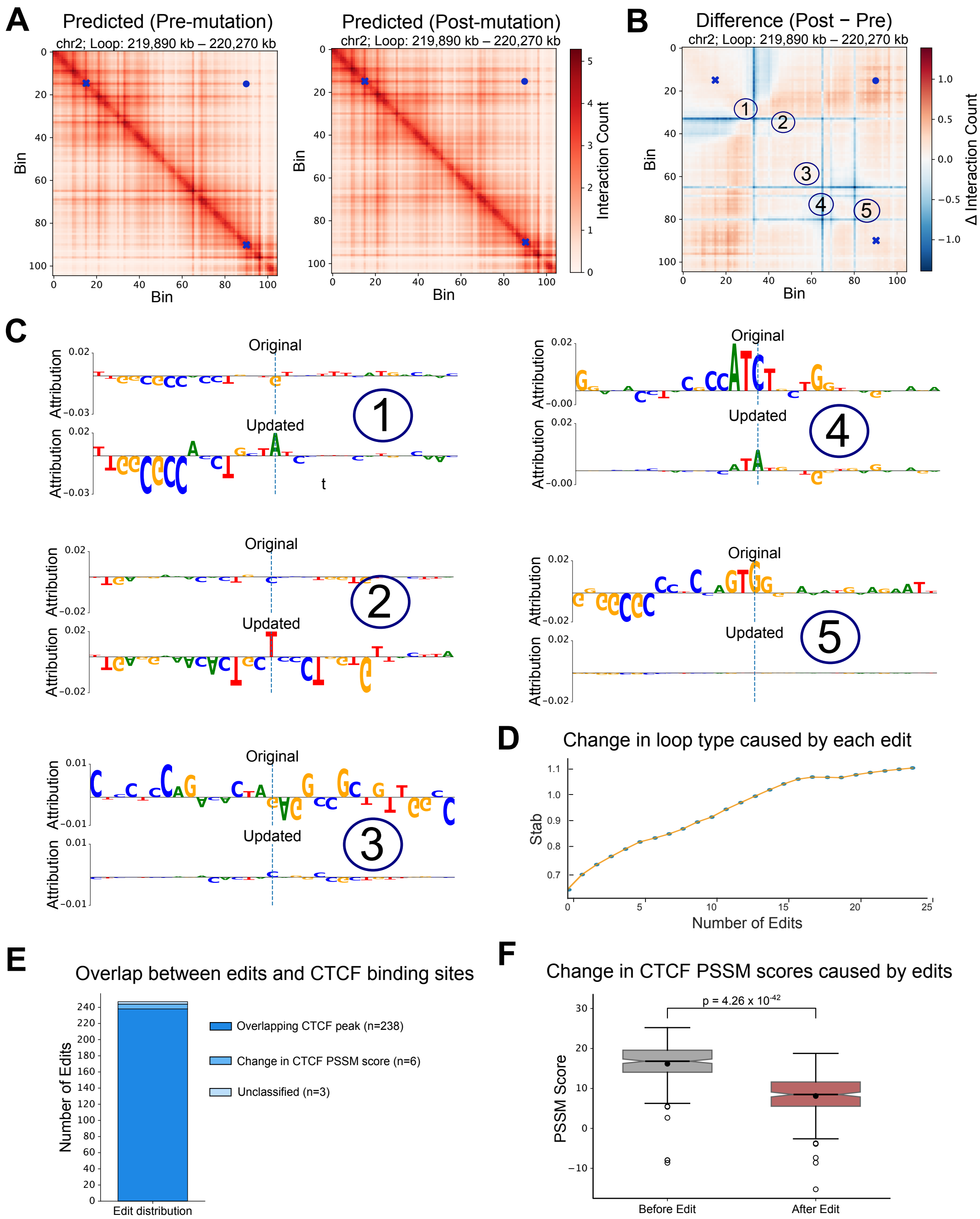

Figure S8: **Fewer intra-loop CTCF sites lead to more stable loop domain.** **A**, Example of a region from the test set where total of five edits are proposed to make a loop more stable. **B**, The difference in predicted contact maps caused by all five proposed edits. **C**, Attribution scores before and after each edit was introduced in the sequence. **D**, Line plot showing change in the optimization function (*Stab*) as a function of number of proposed edits. **E**, Proportion of edits, proposed for symmetric loops in the test dataset, that overlap with CTCF regions. **F**, Change in CTCF PSSM scores caused by the edits across all symmetric loops in the test data. Significance is calculated using Wilcoxon signed-rank test.

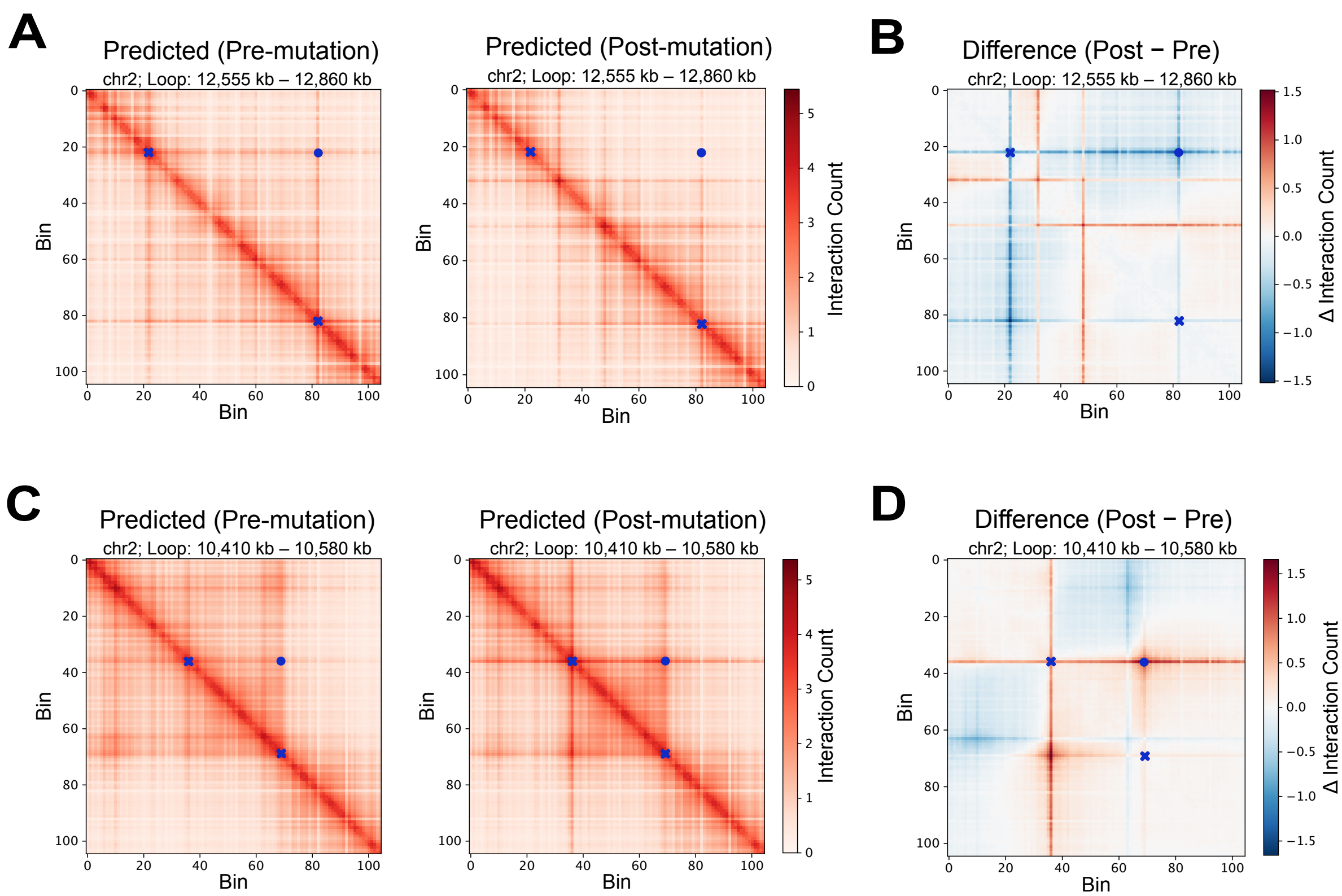

Figure S9: **Detected antipattern on boundary regions if no masking is applied.** **A**, Example of a region from the test set where additional edits on the boundaries are proposed to make a loop more stable. **B**, The difference in predicted contact maps caused by the proposed edits. **C**, Example of a region from the test set where additional edits on the boundaries are proposed to make a loop more extruding. **D**, The difference in predicted contact maps caused by the proposed edits.

Supplementary Tables

Table S1: Edits predicted by our framework to alter loop anchoring and stripe symmetry overlap with known SNPs.

Chrom	Position	SNP ID	Change in Loop Type
chr19	14184412	rs1352588834	Asymmetric to Symmetric
chr19	15272625	rs961480933	Asymmetric to Symmetric
chr19	15273396	rs1403803265	Asymmetric to Symmetric
chr19	41933686	rs566412227	Asymmetric to Symmetric
chr19	56840222	rs1339965976	Asymmetric to Symmetric
chr2	103068666	rs1233062627	Asymmetric to Symmetric
chr2	174313763	rs1406762974	Asymmetric to Symmetric
chr2	236679937	rs1463496071	Asymmetric to Symmetric
chr6	11578350	rs1360966452	Asymmetric to Symmetric
chr6	20092481	rs148095721	Asymmetric to Symmetric
chr6	38941160	rs571045563	Asymmetric to Symmetric
chr6	41754317	rs1369265027	Asymmetric to Symmetric
chr6	166263711	rs540744523	Asymmetric to Symmetric
chr19	4186582	rs1469806546	Symmetric to Asymmetric
chr19	11593584	rs752861056	Symmetric to Asymmetric
chr19	15981885	rs1423089126	Symmetric to Asymmetric
chr19	49979697	rs1463648878	Symmetric to Asymmetric
chr19	52111363	rs987138279	Symmetric to Asymmetric
chr2	86448408	rs749579	Symmetric to Asymmetric
chr2	178539127	rs985932703	Symmetric to Asymmetric
chr6	29802794	rs994405403	Symmetric to Asymmetric
chr6	30646925	rs1363038402	Symmetric to Asymmetric
chr6	40296317	rs989998410	Symmetric to Asymmetric
chr6	55845615	rs994144485	Symmetric to Asymmetric
chr6	55845622	rs1398978912	Symmetric to Asymmetric
chr6	57178837	rs1230360127	Symmetric to Asymmetric

Table S2: Edits predicted by our framework to alter loop stability overlap with known SNPs.

Chrom	Position	SNP ID	Change in Loop Type
chr19	28963962	rs1024259702	Stable to Extruding
chr19	29132131	rs990357913	Stable to Extruding
chr19	29350516	rs959578078	Stable to Extruding
chr19	29360632	rs1200260765	Stable to Extruding
chr19	41488726	rs1225557199	Stable to Extruding
chr19	41497265	rs770247931	Stable to Extruding
chr19	41571108	rs891638207	Stable to Extruding
chr2	46363023	rs1239033001	Stable to Extruding
chr2	57644490	rs1388982439	Stable to Extruding
chr2	68022148	rs1335644990	Stable to Extruding
chr2	84300968	rs1299983807	Stable to Extruding
chr2	84517811	rs572069114	Stable to Extruding
chr2	173091262	rs984242323	Stable to Extruding
chr6	24596304	rs377520480	Stable to Extruding
chr6	24596338	rs1172584687	Stable to Extruding
chr6	24596361	rs138139227	Stable to Extruding
chr6	24596538	rs755928541	Stable to Extruding
chr6	24667267	rs1396785046	Stable to Extruding
chr6	51097101	rs1043196577	Stable to Extruding
chr6	72752943	rs1253717685	Stable to Extruding
chr6	72761593	rs974958979	Stable to Extruding
chr6	100094035	rs1487895477	Stable to Extruding
chr6	100116418	rs890808485	Stable to Extruding
chr6	105142621	rs1299177991	Stable to Extruding
chr6	110500832	rs578166434	Stable to Extruding
chr6	121427160	rs748024905	Stable to Extruding
chr6	122001377	rs1280058946	Stable to Extruding
chr6	124028935	rs973702849	Stable to Extruding
chr6	130938936	rs1486587060	Stable to Extruding
chr6	153795995	rs1003036770	Stable to Extruding
chr6	161920069	rs1364562969	Stable to Extruding
chr6	161920813	rs890713448	Stable to Extruding
chr19	1000246	rs1335548967	Extruding to Stable
chr19	1001836	rs577021345	Extruding to Stable
chr19	1626597	rs1356861490	Extruding to Stable
chr19	3327143	rs933235088	Extruding to Stable
chr19	10380296	rs1248762287	Extruding to Stable
chr19	10594423	rs772359649	Extruding to Stable
chr19	14613354	rs970832964	Extruding to Stable
chr19	39369651	rs200035722	Extruding to Stable
chr19	40970424	rs962747189	Extruding to Stable

Continued on next page

Chrom	Position	SNP ID	Change in Loop Type
chr19	45831508	rs535218778	Extruding to Stable
chr19	47019264	rs1384204628	Extruding to Stable
chr19	47735125	rs535868842	Extruding to Stable
chr19	49249663	rs28400017	Extruding to Stable
chr19	49249858	rs1316792237	Extruding to Stable
chr19	49729472	rs570591062	Extruding to Stable
chr19	49769797	rs1350743534	Extruding to Stable
chr2	11925230	rs1169425282	Extruding to Stable
chr2	73089842	rs1422759286	Extruding to Stable
chr2	85659257	rs995889710	Extruding to Stable
chr2	220341168	rs1427008570	Extruding to Stable
chr2	220406445	rs1199091122	Extruding to Stable
chr2	241535262	rs565982387	Extruding to Stable
chr6	3404354	rs1182997219	Extruding to Stable
chr6	31335134	rs1473229512	Extruding to Stable
chr6	31430752	rs3132090	Extruding to Stable
chr6	37027188	rs1427730174	Extruding to Stable
chr6	39271138	rs1329248215	Extruding to Stable
